# Inhibitory Effect of 7-Demethoxytylophorine on *Penicillium italicum* and its Possible Mechanism

**DOI:** 10.3390/microorganisms7020036

**Published:** 2019-01-26

**Authors:** Chuying Chen, Wenwen Qi, Xuan Peng, Jinyin Chen, Chunpeng Wan

**Affiliations:** 1 Jiangxi Key Laboratory for Postharvest Technology and Nondestructive Testing of Fruits & Vegetables, Collaborative Innovation Center of Postharvest Key Technology and Quality Safety of Fruits and Vegetables, Jiangxi Agricultural University, Nanchang 330045, China; cy.chen@jxau.edu.cn (C.C.); qwwbaobei@163.com (W.Q.); 2 Pingxiang University, Pingxiang 337055, China; pengx1104@163.com

**Keywords:** 7-demethoxytylophorine, antifungal activity, *Penicillium italicum*, membrane integrity, energy deficit

## Abstract

7-demethoxytylophorine (DEM) is a phenanthroindolizidine alkaloid, which is reported to be effective in inhibiting leucocytes and regulation of human immunity. However, few studies reported the inhibitory effect of DEM against plant-pathogenic fungi, particularly postharvest pathogen *Penicillium italicum* (*P. italicum*). Current studies have investigated the antifungal activity of DEM through membrane damage and energy deficit in *P. italicum*. The results showed that the DEM potentially inhibits the growth of *P. italicum* in a dose-dependent manner. In vitro (mycelial growth and spore germination) tests showed great minimal inhibitory concentration (MIC) (1.56 µg mL^−1^) and minimum fugicide concentration (MFC) (6.25 µg mL^−1^). Microscopic analyses showed that mycelial morphology of *P. italicum* was severely damaged following DEM treatment. Moreover, relative electrical conductivity and lysis ability assays showed that DEM treatment aids in destroying the integrity of plasma membranes that deplete reducing sugars and soluble proteins. The activity of malate dehydrogenase (MDH) and succinate dehydrogenase (SDH) demonstrated that DEM led to the disruption of TCA cycle in *P. italicum* mycelia. The results of this study led us to conclude that, DEM could be used as a natural antifungal agent for controlling postharvest blue mold disease of citrus fruits caused by *P. italicum*.

## 1. Introduction

Generally, 10%–30% of citrus fruits loss is caused by blue mold disease in the world; however, in China the loss even more extensive, reaching 30%–50%. Blue mold disease of citrus fruits is caused by *Penicillium italicum* (*P. italicum*); it is cosmopolitan and causes a heavy postharvest loss in the citrus industry of China [1]. There is a limited number of effective synthetic fungicides (imazalil, prochloraz, and triazolone, etc.) that can significantly inhibit the growth of *P. italicum* after it infects citrus fruits. With the onset of awareness about public health due to the excessive use of chemical fungicides and residues retained on citrus fruits, as environmental pollution, led investigators to find alternative strategies for reducing postharvest decay and maintaining fruit quality without health hazards [2,3,4]. Compared to chemical fungicides, plant-derived natural compounds bear no or less toxicity and are generally found to be safe, along with potential antifungal properties to control postharvest fungal rotting of fresh agricultural products [5,6]. Huge numbers of studies have reported the antifungal effects of natural antifungal compounds for controlling postharvest blue mold caused by *P. italicum* that seriously deteriorates citrus crop [7,8,9,10,11].

**Figure 1:**
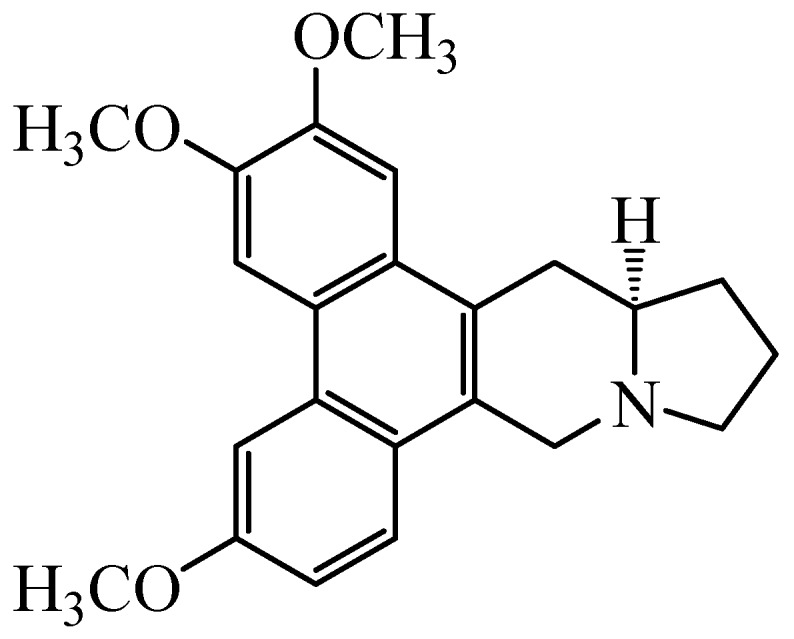
Structure of 7-Demethoxytylophorine (DEM).

An alkaloid 7-Demethoxytylophorine (DEM) (Figure 1) isolated from the rhizomes of *Cynanchum atratum* Bunge, is well known due to its diverse biological activities including hypolipidemic, anti-oxidant, and anti-inflammatory. The *C. atratum* plant has extensively been used in traditional Chinese medicines to cure inflammatory diseases such as hectic fevers, postpartum asthenia, nephritis, skin inflammations, and ulceration [12,13,14]. Recent phytochemical screening of *C. atratum* proved that various anti-inflammatory, anti-fungal, anti-allergic, anti-cancer, immunoregulatory, and anti-TMV activities are mainly due to the presence of C-21 steroidal glycosides [15,16,17,18,19,20]. Recently our lab reported that the ethanolic extracts made from rhizomes of *C. atratum* has strong antioxidant and antifungal activity working against *P. italicum*, and hence shows good preservation effect on “Newhall” navel oranges and “Xinyu” mandarins [16,21,22]. There exists hardly any reports on the possible antifungal mechanisms of DEM on postharvest pathogenic fungus on citrus fruits, especially those involving *P. italicum* that infect fresh citrus fruits to reduce postharvest shelf-life. Keeping in view such huge production losses, we aimed to evaluate the antifungal activity of DEM against *P. italicum* in vitro. Moreover, focus on the possible mechanisms involving morphological changes, plasma membrane permeability, cellular inclusion leakage, and energy deficit in *P. italicum* shall also be studied.

## 2. Materials and Methods

### 2.1. Chemicals

DEM used in this study was extracted and isolated from *C. atratum* in the Jiangxi key laboratory for postharvest technology and nondestructive testing of fruits & vegetables at Jiangxi Agricultural University (Nanchang, China) with the purity of 96.7% [13], and dissolved in 50% acetone to prepare a final optimum concentration of 1 mg mL^−1^ as a stock solution.

### 2.2. Fungal Pathogen and Medium

The isolated *P. italicum* inoculum was obtained from an infected citrus fruit with typical blue mold symptoms and identified on the basis of DNA sequencing. The rDNA-ITS was amplified using the universal primers of ITS1 (5′-TCCGTAGGTGAACCTGCGG-3′) and ITS4 (5′-TCCTCCGCTTATTGATATGC-3′) performed by the National Center for Agricultural Culture Collection (NCACC, Beijing, China). The pure culture of *P. italicum* was grown on potato dextrose agar (homemade PDA: 200 g peeled potatoes, 20 g glucose, 18 g agar powder and 1 L distilled water) medium at 27 ± 1 °C for 7 days and maintained at 4 °C after full growth.

### 2.3. Antifungal Activity Assays

The antifungal activity of DEM was determined by the mycelial growth inhibition assay following the method reported by our previous study [23]. Briefly, the stock solution of DEM (1 mg mL^−1^) was diluted with pure water to obtain the initial concentrations of 0, 7.8, 15.6, 31.3, 62.5 and 125 μg mL^−1^ and diluted 10 times with PDA for obtaining the final concentrations of 0, 0.78, 1.56, 3.13, 6.25 and 12.5 µg mL^−1^. The mycelial disks (6 mm in diameter), cut from the periphery of a seven-day-old culture using a stainless steel punch, was placed in the center of each petri dish (90 mm in diameter). Then, all plates were incubated at 27 ± 1 °C for about 7 days. Four replicates were used per treatment and the experiment was carried out at two separate times. Mycelial growth inhibition (MGI) of DEM treatment against control was calculated using the following equation:

(1) $$Mycelial\;growth\;inhibition\;\left(MGI,\;\%\right)\;=\frac{D_{c}-D_{t}}{D_{c}-D_{i}}\;\times 100$$

where *D_c_* and *D_t_* are the mean colony diameter of control and treated sets, respectively; *D_i_* is the initial colony diameter of mycelial PDA disks.

The minimal inhibitory concentration (MIC) was defined as the lowest DEM concentration that completely inhibited the growth of *P. italicum* after 48 h of incubation at 27 ± 1 °C. The minimum fungicidal concentration (MFC) defined as the lowest concentration of DEM with no visible fungal growth on a PDA plate after five days of incubation at 27 ± 1 °C [24].

The antifungal stability of DEM was determined by the spore germination inhibition assay according to the method described by Dou et al. [25] with some minor modifications. Briefly, the tested *P. italicum* strain was grown on a PDA plate medium at 27 ± 1 °C for 7 days. The plates were then immersed with 5 mL sterile water and gently scraped with a sterile batten to obtain a spore suspension (1×10^6^ spores/mL). DEM was transferred to sterile concavity slides with potato dextrose broth (PDB, containing 20 μL of spore suspensions) for obtaining the final concentrations of 0, 0.78, 1.56, 3.13, and 6.25 µg mL^−1^. All the culture slides were placed on moist sterile paper in petri plates, sealed with parafilm to avoid water evaporation and competitor. For incubating at 27 ± 1 °C for 12 h, each slide was fixed with acid fuchsine solution to stop further germination. Approximately 100 spores within each replicate were observed using a reversed biological microscope (Olympus CKX53, Tokyo, Japan). Spore germination inhibition (SGI) of DEM treatment against control was calculated using the following equation:

(2) $$Spore\;germination\;inhibition\;\left(SGI,\;\%\right)=\frac{G_{c}-G_{t}}{G_{c}}\times 100$$

where *G_c_* and *G_t_* are the mean number of germinated spores of control and treated slides, respectively. Four replicates were used per treatment and the experiment was carried out with two separate times.

### 2.4. Determination of Membrane Permeability

The determination of relative electric conductivity was carried out using a ST3100c/F electrical conductivity meter (Ohaus Co., Ltd., Parsippany, NJ, USA). After 48 h of shake inoculating in PDB at 27 ± 1 °C, DEM solution at various concentrations (0, MIC, 2 MIC and MFC) was added and inoculated for 24 h. The supernatant was collected by centrifugation at 6000× *g* for 20 min and used for the assay of extracellular electric conductivity. The relative electric conductivity from the DEM-treated and control group was calculated using the following equation:

(3) $$Relative\;electric\;conductivity\;(REC,\;\%)=\frac{C_{i}-C_{0}}{C_{0}}\times 100$$

where *C*_0_ and *C_i_* are the electric conductivity determined at the treatment of 0 h and 24 h, respectively. Four replicates were used per treatment and the experiment was carried out with two separate times.

The assay of cell lysis rate from the DEM-treated and control group was determined by spectrophotometry. After 24 h of incubation, the supernatant was collected and used to measure absorbance at 650 nm (Shimadzu UV-2600, Tokyo, Japan). The cell lysis rate was expressed as the difference between final and initial absorbance at 650 nm and calculated using the following equation:

(4) $$Cell\;lysis\;rate\;\left(CLR,\;\%\right)=\frac{A_{i}-A_{0}}{A_{0}}\times 100$$

where *A*_0_ and *A_i_* are the absorbance determined at the treatment of 0 h and 24 h, respectively. Four replicates were used per treatment and the experiment was carried out with two separate times.

### 2.5. Assay for Reducing Sugars and Soluble Proteins Content

For reducing sugar content assay, about 0.5 g of freeze-dried mycelia from the DEM-treated and control PDB were homogenized in 8 mL of distilled water, and then extracted by boiling water bath for 15 min. Subsequently, both extracts of mycelia and suspensions were cooled at room temperature for 10 min, diluted with distilled water to 250 mL, and then added to 2.5 mL of 10% (*v*/*v*) lead acetate. After 15 min, the reaction system was filtered with a Buchner funnel after adding 0.5 g of crystal violet oxalate to remove redundant lead acetate. Subsequently, 2.0 mL of filtrate was heated for 5 min in a boiling water bath after mixing with 0.5 mL of anthrone reagent and 5.0 mL of H_2_SO_4_, and then quickly cooled in an ice bath. The reducing sugar content in mycelia was measured at 620 nm to calculate reducing sugar content (mg g^−1^ frozen weight) from the standard curve using glucose as a standard.

About 0.5 g of freeze-dried mycelia from the DEM-treated and control PDB were homogenized in 5 mL of phosphate buffer (0.05 mol L^−1^, pH 7.2) and centrifuged at 4000× *g* for 15 min at 4 °C. A total of 0.2 mL of supernatant was added to 10 mL of coomassie brilliant blue (G-250). After incubation for 20 min, the tested sample was monitored for absorbance at 595 nm using a M2 Multiscan Spectrum microplate reader (Molecular Devices Corporation, Sunnyvale, CA, USA). The soluble proteins content in mycelia was expressed as mg g^−1^ frozen weight.

### 2.6. Assay for the Release of Reducing Sugars and Soluble Proteins

The release of reducing sugar and soluble protein was measured according to the method described by Jing et al. [26] with some modifications. Briefly, *P. italicum* suspensions from 100 mL PDB were collected by centrifugation at 4000× *g* for 30 min, washed three times with sterilized distilled water, and resuspended in 100 mL phosphate buffer (0.1 mol L^−1^, pH 7.0). After that, the suspensions were treated with DEM at various concentrations (0, MIC, 2MIC and MFC) for 0 and 24 h. Then, 0.2 mL of supernatant was monitored for the absorbance of 620 nm and 595 nm using a M2 Multiscan Spectrum microplate reader (Molecular Devices Corporation, Sunnyvale, CA, USA). The release of reducing sugar and soluble protein was expressed as OD_620_ mL^−1^ and OD_595_ mL^−1^, respectively.

### 2.7. Assay for Activities of MDH and SDH

Control and DEM-treated mycelia were homogenized in 8 mL of ice-cold phosphate buffer (0.05 mol L^−1^, pH 7.2) and centrifuged at 10,000× *g* for 15 min at 4 °C. The activities of MDH and SDH in the supernatant were measured using commercially available kits purchased from Nanjing Jiancheng Bio-engineering Institute (Nanjing, China) according to the manufacturer’s instructions. The activities of MDH and SDH were detected at 340 nm and 600 nm (Shimadzu UV-2600, Japan), respectively. All tests were performed in three replicates.

### 2.8. Statistical Analysis

All experimental data were SPSS version 17.0 (SPSS Inc., Chicago, IL, USA). Data from assay of MGI, SGI, REC, CLR, contents of reducing sugar and soluble protein, activities of MDH and SDH were analyzed with a Student’s *t*-test, and significant differences were analyzed using one-way analysis of variance (ANOVA) at the 5% level.

## 3. Results

### 3.1. Mycelial Growth and Spore Germination

The inhibitory effect of DEM on mycelial growth of *P. italicum* is presented in Table 1. The mycelial growth of *P. italicum* on PDA medium was significantly inhibited by DEM in a dose-dependent manner (*p* < 0.05). DEM inhibited approximately 30% of the *P. italicum* mycelial growth at comparatively low concentration of 0.78 µg mL^−1^. DEM at 3.13 µg mL^−1^ inhibited over four fifths of the *P. italicum* mycelial growth, with the mycelial growth inhibition of 82.43%. In particular, the mycelial growth of *P. italicum* was completely inhibited by DEM at the concentration of 6.25 µg mL^−1^. Therefore, MIC and MFC of DEM was 1.56 µg mL^−1^ and 6.25 µg mL^−1^, respectively.

The effect of DEM on spore germination of *P. italicum* in PDB is well elaborated (Table 1). The spore germination was significantly inhibited by increasing DEM concentration (*p* < 0.05). When the concentration of DEM reached 6.25 µg mL^−1^, less than 5% of *P. italicum* spores germinated.

### 3.2. Light Microscopy

**Figure 2:**
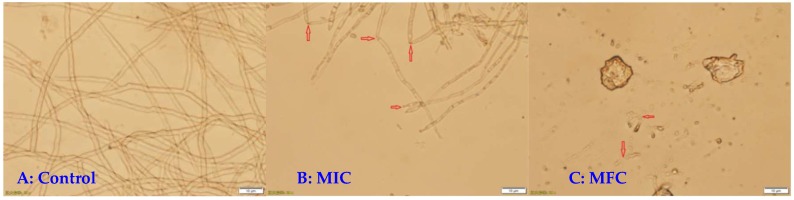
Effect of DEM on hyphal morphology of *P. italicum*. normal mycelial morphology (**A**), mycelia treated with MIC of DEM (**B**), mycelia treated with MFC of DEM (**C**).

Light microscopy of the control *P. italicum* mycelia grown on PDA showed normal and homogeneous mycelial morphology (Figure 2A). By contrast, the mycelia treated with MIC of DEM showed a massive distortion and abnormal enlargement of growing point (Figure 2B), whereas the DEM-treated mycelia at MFC significantly checked the spore germination and altered the hyphal morphology of *P. italicum*, including loss of linearity, breakage, malformation, and agglomeration (Figure 2C). Obviously, DEM treatment resulted in damage to *P. italicum* mycelia.

### 3.3. Membrane Permeability

**Figure 3:**
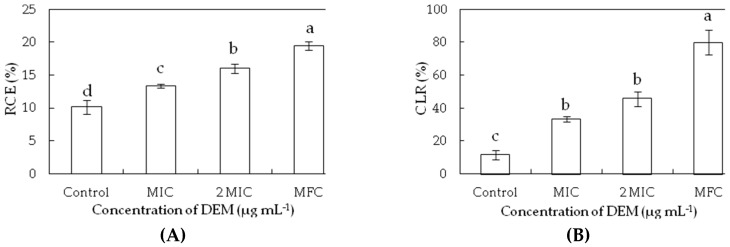
Effect of DEM on relative electric conductivity (**A**) and cell lysis rate (**B**) of *P. italicum*.

The relative electric conductivity and cell lysis rates were used for determining the damage of membrane permeability. The relative electric conductivity and cell lysis rates were increased after exposure to increasing concentrations of DEM compared to the controls (*p* < 0.05) (Figure 3). The relative electric conductivity increased from 10.13% for the control to 13.34%, 15.98% and 19.42% *P. italicum* suspensions with the treatment of DEM at MIC, 2MIC, and MFC at 24 h, respectively, which indicated that the higher concentrations of DEM caused more serious damage to plasma membrane (Figure 3A). At increased DEM concentrations, the cell lysis rate of *P. italicum* suspensions increased by 2.87, 3.95, and 6.90 times compared to that at 24 h for control (11.56%), showing that the higher concentration of DEM led to a significant growth after triggering an accelerated cell disruption in *P. italicum* suspensions.

### 3.4. Intracellular and Extracellular of Cytoplasmic Constituents

**Figure 4:**
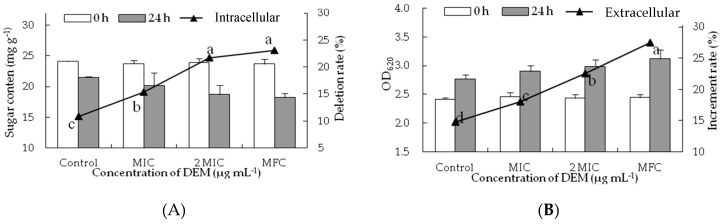
Effect of DEM on sugar content from intracellular (**A**) and extracellular (**B**) of *P. italicum*.

To confirm the membrane-peroxidization effects of DEM on intracellular and extracellular, cell constituents (reducing sugar and soluble protein) were evaluated. DEM treatment significantly hampered the biosynthesis and production of inclusions in *P. italicum* mycelia. As shown in Figure 4A, the reducing sugar content of DEM treated *P. italicum* mycelia at MIC, 2MIC and MFC were 20.12 ± 2.12 mg g^−1^, 18.73 ± 0.46 mg g^−1^ and 18.21 ± 0.71 mg g^−1^, respectively, much lower than that in control samples after the incubation of 24 h (21.50 ± 0.12 mg g^−1^). The reducing sugar content of *P. italicum* mycelia continuously decreased as the gradient ascent of DEM (*p* < 0.05), whereas there were no apparent differences between 2 MIC and MFC of DEM treatment. Figure 4B showed the effect of the release of reducing sugar when *P. italicum* suspensions were treated with DEM at different concentrations (MIC, 2MIC and MFC). The OD_620_ of *P. italicum* suspensions treated with DEM were dramatically increased after 24 h of exposure. The increment rate of OD_620_ treated with DEM at MFC was 27.47%, which was significantly higher (*p* < 0.05) than that in 2 MIC (22.59%), MIC (18.03%) or control (14.48%). Those results suggested that the cytoplasmic constituents of *P. italicum* were infiltrated into the lipid medium after DEM treatment, thus the extracellular reducing sugar content in *P. italicum* suspensions was much higher than that of the control.

**Figure 5:**
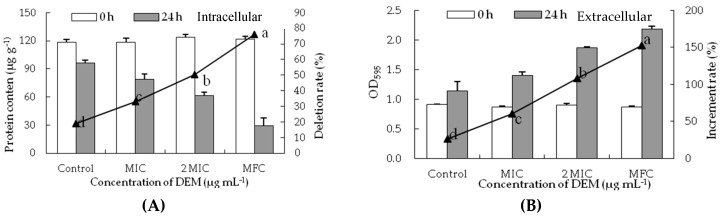
Effect of DEM on protein content from intracellular (**A**) and extracellular (**B**) of *P. italicum*.

Similarly, the soluble protein content of *P. italicum* mycelia significantly decreased with the different concentrations of DEM treatment (Figure 5A). The soluble protein content of DEM treated *P. italicum* mycelia at MIC, 2MIC, and MFC were 20.12 ± 2.12 mg g^−1^, 18.73 ± 0.46 mg g^−1^ and 18.21 ± 0.71 mg g^−1^, respectively, which were much lower than that in control samples (21.50 ± 0.12 mg g^−1^) after the incubation of 24 h. The release of soluble protein of *P. italicum* suspensions significantly increased with the different concentrations of DEM treatment (Figure 5B). The increment rate of OD_595_ treated with DEM at MFC was 152.43%, which was increased by 5.81, 2.53 and 1.41 times compared to that at 24 h for control (26.24%), MIC (60.23%), and 2MIC (108.00%), respectively.

### 3.5. Activities of MDH and SDH

**Figure 6:**
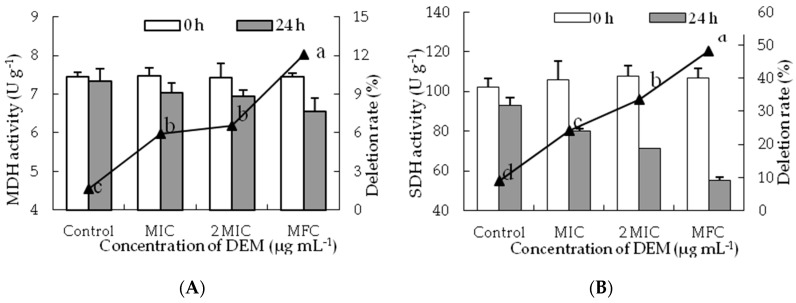
Effect of DEM on the activities of MDH (**A**) and SDH (**B**) of *P. italicum*.

The MDH activity of treated *P. italicum* mycelia decreased with increasing DEM concentrations and those in the control samples still remained stable during the inoculation at 24 h (Figure 6A). After 24 h, the MDH activity of DEM treated *P. italicum* mycelia at MIC, 2MIC, and MFC were decreased with the levels reaching 7.03 ± 0.26 U g^−1^, 6.93 ± 0.16 U g^−1^ and 6.54 ± 0.35 U g^−1^. However, the MDH activity in control samples was 7.33 ± 0.32 U g^−1^, which were much higher than that of DEM treated *P. italicum* mycelia at 2 MIC and MFC (*p* < 0.05).

SDH activity decreased constantly after incubation for 24 h and a significant difference was shown between DEM treatment and control group (Figure 6B). After the incubation of 24 h, the SDH activities of DEM treated *P. italicum* mycelia at MIC, 2 MIC and MFC were 80.23 ± 1.14 U g^−1^, 71.51 ± 0.18 U g^−1^ and 55.35 ± 1.53 U g^−1^, whose deletion rate were 24.26%, 33.47% and 48.12% than that in control samples, respectively.

## 4. Discussion

Various plant-derived antifungal active compounds such as cinnamaldehyde, citral, citronellal, geraniol, octanal, pinocembrin, and different essential oils have extensively been studied for their potential to target fungal pathogens in agricultural products [7,25,27,28,29,30,31,32,33]. The natural phytochemicals that bear strong antifungal properties can help reduce the consumption of synthetic fungicides and successfully eradicate outbreaks of fungal diseases, maintain fruit quality, safeguard the environment, and promote higher biodegradability and reduced toxicity [3,6,34]. The DEM is said to be a natural alternative to the synthetic fungicides that control postharvest blue mold disease of citrus fruits caused by *P. italicum* [13].

Current in vitro study showed that both the mycelial growth and spore germination of *P. italicum* were decreased in a dose-dependent manner, and significantly decreased treatment by MIC (1.56 µg mL^−1^) and MFC (6.25 µg mL^−1^) (Table 1). Moreover, DEM promotes prominent antifungal activity against *P. italicum* on PDA plates and liquid media. A similar study reported about 90% MGI against *Alternaria solani* in tomatoes, *Cercospora arachidicola* in peanuts, *Physalospora piricola* in apples, and *Cladosporium cucumerium* in cucumbers when treated with DEM at 50 µg mL^−1^ [35]. The DEM showed the lowest MIC value against *P. italicum* in comparation with pinocembrin (400 µg mL^−1^), octanal (64.11 µg mL^−1^), and citral (76.12 µg mL^−1^) in previous studies [8,9,11]. DEM treatment (6.25 µg mL^−1^) significantly reduced the spore germination (4.37%) of *P. italicum* brought lower than 5%. Few natural antifungal agents such as octanal and citral, showed a complete inhibition of mycelial growth of *P. italicum* when added to the PDA medium at 0.5 µL mL^−1^ [8,9]. Accordingly, green mold caused by *P. digitatum* on citrus fruit, the spore germination, and mycelial growth were also completely inhibited by the addition of 0.5 µL mL^−1^ octanal, 3.2 µL mL^−1^ citronellal and 8.0 µL mL^−1^ cinnamaldehyde to PDA plates [25,26,31].

Microscopic analyses showed an obvious difference between the DEM-treated and control mycelia of *P. italicum*. The DEM treated mycelia were observed and MIC formed were draped and inflated at growing point, whereas those exposed to DEM (6.25 µg mL^−1^) treatment showed MFC were aggregated and collapsed. DEM (6.25 µg mL^−1^) cause heavy damage to mycelium plasma membrane of *P. italicum*, leading to spillage of intracellular macromolecular cytoplasmic components into the extracellular suspensions through damaged sites. These findings indicated that DEM can induce damage to the mycelium plasma membrane of *P. italicum* and is mimicking the reports for various other antifungal agents like citral, octanal, and pinocembrin that kills *P. italicum* and reduces blue mold in citrus fruits [8,9,11,36]. Many related studies stated that *P. digitatum* induced green molds in citrus fruits and were also reduced in similar mechanisms by using α-terpineol, β-carbolines, citral, octanal, and citronellal [26,30,31,37]. *Geotrichum citri-aurantii* causing sour rot in citrus fruits treated with α-terpineol, citral, octanal, cinnamaldehyde, and thyme oil were also following the same pattern of actions [27,29,32]. Grey mold caused by *Botrytis cinerea* in blueberries, tomatoes, strawberries, and table grapes were also successfully treated by using â-carbolines, p-coumaric, phenazine-1-carboxylic acid (PAC), and pterostilbene [37,38,39,40], showing similar modes of action to stop deterioration of the fruits. *P. expansum*-caused blue mold in kiwifruit treated with quercetin, cinnamaldehyde, and citral shows great success in lowering crop damage [41,42]. The changes in *P. italicum* mycelia in the current study may be attributed to amplified membrane permeability, and they usually mean the leakage of cell constituents including reducing sugar, soluble proteins, and sugars in cell metabolism (Figure 3, Figure 4 and Figure 5).

Reducing sugar and soluble protein are two main components of cell cytoplasm, and any decline in their levels usually suggests a reduction in membrane stability to intracellular mycelia and an increased permeability to extracellular suspensions [9,36,38,39]. In the present study, DEM significantly decreased the contents of reducing sugars and soluble protein of *P. italicum* mycelia (Figure 4A and Figure 5A). Meanwhile, increasing concentration of DEM led to a rapid decrease in concentration of these sugars and proteins at 620 nm and 595 nm in *P. italicum* suspensions (Figure 4B and Figure 5B). Moreover, DEM actively disrupts cellular homeostasis and disturbs normal biosynthetic pathways of energy production by significantly reducing the contents of cell constituents. Literature reported similar findings that described 2,4-Diacetylphloroglucinol (2,4-DAPG), citral, octanal, and pinocembrin have inhibitory effects on *P. italicum* growth [8,9,11,36,43]. These findings have implied that membrane stability can be a crucial anti-fungal target by DEM.

Natural phytochemicals not only disrupted the stability of cell membranes, but also hampered energy transduction procedures and caused the leakage of ATP from fungal hyphae [3,44]. To find an in-depth mechanism of possible antifungal actions of DEM involves a strong influence on the energy metabolism of *P. italicum*. Furthermore, results depicted the process of cell membrane damage was enslaved to energy deficit of *P. italicum* caused by DEM treatment (Figure 6), which is thought of as the possible antifungal mechanism of DEM against *P. italicum* by distraction of tricarboxylic acid cycle (TAC) of hyphal cells, leading to energy deficiency and cell membrane damage ultimately accelerating cell death. The activities of MDH and SHD in *P. italicum* mycelia declined sharply following DEM exposure (Figure 6A,B), indicating the severe shortage of energy supply results in the cellular metabolic disorder in *P. italicum* mycelium along with generation of efflux of reactive oxygen species (ROS) imbalance and ultimately cell apoptosis. These results are in conformity with recent reports describing cinnamaldehyde effects on *P. italicum*, *P. expansum* and *A. alternata* [7,28,45], citral effects on *P. digitatum* [30], tea tree oil effects on *Botrytis cinerea* [33,46], and l-glutamate effects on *A. alternate* [47]; further confirmed TCA pathway in *P. italicum* mycelia was disturbed by DEM. However, further in-depth studies are still needed to explain the molecular antifungal mechanism of DEM treatment against *P. italicum*-caused postharvest blue mold in citrus fruits.

## 5. Conclusions

This is the pioneer report in which DEM treatment was proved to be effective against *P. italicum.* The in vitro inhibitory effects of mycelial growth and spore germination of *P. italicum* were increased in a concentration-dependent DEM, where MIC and MFC were 1.56 µg mL^−1^ and 6.25 µg mL^−1^, respectively. Microscopic observations showed that mycelial morphology was seriously damaged after DEM treatment. Moreover, DEM might exert its antifungal activity via membrane-targeted mechanism with increased membrane permeability, disrupted membrane stability causing the large leakage of cytoplasmic inclusions, and the severe deficit of energy, finally resulting in cell death of *P. italicum*. It is stated that DEM would be an effective and novel alternative to chemical fungicides for controlling postharvest blue mold of citrus fruit. Later studies are required to explain the antifungal mechanism of DEM against *P. italicum* at the molecular level.

## Figures and Tables

**Table 1:** Effect of different DEM concentrations on mycelial growth and spore germination of *P. italicum* after incubation at 27 ± 1 °C.

Concentrations (µg mL^−1^)	Mycelial Growth ^1^		Spore Germination ^2^	
	Mycelial Diameter (mm)	MGI (%)	Germination Rate (%)	SGI (%)
0	55.50 ± 1.29 a	0 e	94.77 ± 2.63 a	0 f
0.78	38.25 ± 0.96 b	30.08 ± 2.27 d	71.68 ± 2.42 b	23.36 ± 2.55 e
1.56	24.00 ± 1.41 c	56.77 ± 2.55 c	49.55 ± 3.01 c	47.72 ± 3.18 d
3.13	9.75 ± 1.71 d	82.43 ± 3.08 b	26.16 ± 2.75 d	72.40 ± 2.90 c
6.25	0 e	100 ± 0.00 a	4.37 ± 0.18 e	95.39 ± 0.19 b
12.50	0 e	100 ± 0.00 a	0 f	100 ± 0.00 a

^1^ Mycelial growth was measured after incubation at 27 °C for 7 days. Data presented are the means ± S.E. (*n* = 8). The column with different lowercase letters between different concentrations indicates significant differences according to Duncan’s test (*p* < 0.05). ^2^ Spore germination was determined after incubation at 27 °C for 12 h. DEM: 7-demethoxytylophorine, MGI: Mycelial growth inhibition, SGE: Spore germination inhibition.
